# Endogenous Repair by the Activation of Cell Survival Signalling Cascades during the Early Stages of Rat Parkinsonism

**DOI:** 10.1371/journal.pone.0051294

**Published:** 2012-12-12

**Authors:** Nga-Ping Lui, Liang-Wei Chen, Wing-Ho Yung, Ying-Shing Chan, Ken Kin-Lam Yung

**Affiliations:** 1 Department of Biology, Hong Kong Baptist University, Kowloon Tong, Hong Kong; 2 Institute of Neurosciences, The Forth Military Medical University, Xian, PR China; 3 School of Biomedical Sciences, The Chinese University of Hong Kong, Shatin, New Territories, Hong Kong; 4 Department of Physiology and Research Centre of Heart, Brain, Hormone and Healthy Aging, LKS Faculty of Medicine, The University of Hong Kong, Hong Kong; Hertie Institute for Clinical Brain Research and German Center for Neurodegenerative Diseases, Germany

## Abstract

Here we report a previously unknown self repair mechanism during extremely early stages of rat Parkinsonism. Two important cell survival signaling cascades, Phosphatidylinositol-3 kinases (PI3K)/Akt pathway and extracellular signal-regulated kinase/mitogen-activated protein kinase (ERK/MAPK) pathway, could be responsible for this potential endogenous rescue system. In the 6-hydroxydopamine-lesioned rat, the phosphorylated p44/42 MAPK and its downstream target, the phosphorylated Bad at Ser 112, were up-regulated at post-lesion day 3 and lasted for a couple of weeks. Although the change in the phosphorylated Akt kinase was negligible throughout the studied period, its downstream target, the phosphorylated Bad at 136, was increased from post-lesion day 3 to post-lesion day 14. In the mean time, nestin-positive reactive astrocytes with low levels of brain-derived neurotrophic factor (BDNF) and glial cell line-derived neurotrophic factor (GDNF) appeared at post-lesion day 3 in 6-hydroxydopamine-lesioned rat. BDNF was expressed in both striatum and substantia nigra whereas GDNF was displayed in striatum only. At post-lesion day 14, nestin, BDNF and GDNF expressions were diminished. These neurotrophic factors were believed to initiate the above anti-apoptotic signal transduction cascades as we could see that their expression patterns were similar. The data strongly suggest that there is an endogenous repair effort by evoking the cell survival signaling and possibly via the releases of BDNF and GDNF from nestin-immunoreactive reactive astrocytes. ERK/MAPK pathway was proposed to be the key endogenous neuroprotective mechanisms, particularly in early stages of rat Parkinsonism. However, the self repair effort is only functional within an extremely short time window immediately after onset.

## Introduction

Parkinson's disease is a serious movement disorder and the sine qua non of Parkinson's disease is the massive loss of dopaminergic neurones in the substantia nigra pars compacta [1], [2], [3], [4], [5]. Neuroprotection and restoration of the dopaminergic neurones are the therapeutics strategies against Parkinson's disease in humans. It was reported that apoptosis caused the neuronal degeneration of PD [6], [7]. Moreover, a pro-apoptotic environment could be detected in the nigrostriatal region of Parkinsonism [7]. The apoptotic death of cells was characterized by the expression of oncogenes, including the apoptotic promoter (Bcl-X_S_, Bax and Bad) as well as the apoptotic inhibitors (Bcl-2, Bcl-X_L_) [6], [8]. The balance of the death and the survival signals determined the fate of the cell.

Bad (Bcl-X_L_/Bcl-2 associated death promoter) is a pro-apoptotic member of Bcl-2 family. It can provoke an apoptotic cascade by forming a heterodimer with either Bcl-2 or Bcl-X_L_. The antagonistic effect of Bad on the survival-promoting activity is stronger than the other apoptotic promoters [9]. The phosphorylation of Bad results in releasing Bcl-2 and Bcl-X_L_ and thus inhibiting the apoptotic program. The increase in several phosphorylated kinase, such as Akt and MAPK, also accompany the phosphorylated Bad and enhance cell survival [9], [10], [11].

Akt kinase exerts a direct effect on the cell survival pathway by targeting the pro-apoptotic Bad and mediating the phosphorylation of Bad at Ser 136. Besides the release of Bcl-2 and Bcl-X_L_, Bad creates a binding site to 14-3-3 proteins and allows the inactivation of its apoptotic property [12]. Like Akt pathway, MAPK pathway is considered as a crucial cell survival pathway against the apoptosis. The activation of the p44/42 MAPK (ERK1/2) pathway requires the phosphorylation of threonine and tyrosine residues [13]. ERK1 and ERK2 are homologous isoforms and share similar substrate specificities [14]. The activation of the MAPK kinase can suppress the activity of Bad by inducing the phosphorylation of Bad at Ser 112 [15]. It is suggested that Akt and MAPK signaling pathways converge at Bad and inhibit cell death.

In the present study, the changes in Akt and MAPK signaling pathways during early stages of Parkinson's disease would be explored by using the 6-hydroxydopamine (6-OHDA)-lesioned rat model. We suggested that the re-appearance of nestin-postive reactive astrocytes with the up-regulations of brain-derived neurotrophic factor (BDNF) and glial cell line-derived neurotrophic factor (GDNF) could potentially induce this self repair mechanism.

## Materials and Methods

### Animals

All experiments were conducted with female Sprague-Dawley (SD) rats weighting from 200 to 220 g. The animal experimental protocols performed in this study strictly confirmed to the guidelines of the Animals (Control of Experiments) Ordinance, Department of Health, Hong Kong, and approved by the Committee on the Use of Human and Animal Subjects in Teaching and Research, Hong Kong Baptist University, and also conformed to the Principles of Laboratory Animal Care (NIH publication no. 86-23, revised 1985). All procedures performed were aimed to minimize both animal number and suffering of the animals.

### 6-hydroxydopamine lesion

The striatal 6-OHDA lesion was performed in this experiment [16], [17], [18], [19], [20], [21], [22]. [23]. The SD rats were divided into following groups, i.e. post-lesion day 1 (n = 5), day 3 (n = 5), day 5 (n = 5), day 7 (n = 5), day 14 (n = 5) and control (n = 25). The SD rats were first anesthetized with 2–3 µ1 of sodium pentobarbital (60 mg/kg; i.p.; Saggittal) before the surgery of lesion. 6 ul of a 6-OHDA solution (Sigma; 3 mg/ml) dissolved in ascorbic acid solution (0.2 mg/ml in 0.9% saline) was then stereotaxically injected at point one: Bregma: +0.l4 cm, Medline: −0.26 cm, Dura: −0.5 cm, and point two: Bregma: −0.04 cm, Medline: −0.38 cm, Dura: −0.5 cm in accordance to the atlas of Paxinos and Watson, (1986). Control group was done by administrating 6 ul of 0.9% saline instead of 6-OHDA solution. At above survival dates after 6-OHDA lesion, the rats were administrated with apomorphine (1 mg/ml; i.p.; RBI) for the test of the rotational behaviour [24], [25].

### Western blotting

The animals were sacrificed by decapitation. The brains were immediately removed. The whole striatum and substantia nigra were rapidly dissected and placed on the ice. The target proteins were extracted from each samples protein of the brain. Samples were homogenized with the suggested buffer and then centrifuged at 13,000×g for 30 min at 4°C. The resultant supernatants were collected and frozen at −80°C until analysis. 50 µg of protein sample was used. The sample proteins were resolved in SDS-PAGE gels by electrophoresis and were blotted to nitrocellulose membranes through standard Western blotting protocol [26]. Blocking of nonspecific binding was done by incubating the membranes with 5% non-fat milk for 2 hours at room temperature and then with the primary antibodies in 2% non-fat milk overnight at 4°C. The antibodies used were chicken anti-GDNF (Millipore), sheep anti-BDNF (Millipore), rabbit anti-phosphorylated Akt (Ser 473; Cell Signaling Technology), rabbit anti-Akt (pan; Cell Signaling Technology), mouse anti- phosphorylated p44/42 MAPK (Erk1/2) (Thr202/Tyr204; Cell Signaling Technology), rabbit anti- p44/42 MAPK (Erk1/2; Cell Signaling Technology), rabbit anti-Bad (Ser 136; Cell Signaling Technology) and rabbit anti-Bad (Ser 112; Cell Signaling Technology) in the ratio of 1 to 1000, and rabbit anti-TH (Millipore) and mouse anti-β actin (Millipore) in the ratio of 1 to 3000. After rinsing the membranes with TBS-T and TBS several times, they were incubated with the appropriate horseradish peroxidase [HRP]-conjugated secondary antibodies (1∶4000; Zymed Laboratories Inc.) against the primary antibodies for 1 hour at room temperature. Bands on the membranes were visualized using chemiluminescence detection (ECL Western blotting detection reagents; Amersham Pharmacia). Images of the bands were developed on films (Biomax X-ray film; Kodak).

### BDNF and GDNF ELISA assays

BDNF and GDNF proteins were measured by BDNF E_max_ immunoassay system and GDNF E_max_ immunoassay system (Promega, Madison, WI) according to the protocols of the manufacturer respectively. 50 µg of protein sample was used. The wells of 96-well plate were coated well with the respective monoclonal antibodies and incubated overnight at 4°C. After several washes with TBS-T, Block & Sample 1× buffer was added for the non-specific binding blocking for 1 hour at room temperature, following with the incubation of extracted samples and the standards with the immobilized monoclonal antibodies for 2 hours at room temperature. The incubations of the appropriate polyclonal antibodies and the Anti-IgY HRP conjugates were then done. 3,3′,5,5′-tetramethylbenzidine (TMB) one solution was added for the colour development which was detected by a multi-functional plate reader (Tecan Infinit F200) at 450 nm.

### Immunofluorescence

Immunofluorescence was performed to illustrate the immunoreactivity for nestin (MAB353; Chemicon/Millipore) and tyrosine hydroxylase (TH; MAB318; Chemicon/Millipore). The striatum and substantia nigra sections were incubated overnight with rabbit anti-nestin (1∶500) and mouse anti-TH (1∶2000) in PBS (0.01 M, pH 7.4) with 0.1% Triton-X (Fluka) and 2% normal goat serum (Vector Laboratories Inc.) at room temperature. The sections were then rinsed with PBS and incubated in Alexa 488-conjugated secondary antibody solution which was anti-primary origins IgG (1∶500; Molecular Probes) for 2 hours in dark at room temperature. After being rinsed with PBS, the sections were mounted on clean slides with fluorescent mounting medium (Dako) and covered with coverslips for the examination under the same parameters with laser scan confocal microscope (Olympus fluoview 1000).

### Double immunofluorescence

For characterization of nestin immunoreactive cells, double immunofluorescence was performed in the striatum and substantia nigra sections of the 6-OHDA-treated rats in order to visualize cellular co-localization of nestin with glial fibrillary acidic protein (GFAP; Millipore), GDNF (Millipore) and BDNF (Millipore). The procedures were similar to that of the simple immunofluorescence. The striatum and substantia nigra sections were incubated overnight at room temperature with a mixture of two different antibodies. For nestin/GFAP double labeling and nestin/GDNF double labeling, rabbit anti-nestin (1∶500) with rat anti-GFAP (1∶1000) and rabbit anti-nestin (1∶500) with chicken anti-GDNF (1∶100), were diluted in PBS (0.01 M; pH 7.4) with 0.1% Triton-X and 2% normal goat serum. For nestin/BDNF double labeling, rabbit anti-nestin (1∶500) with sheep anti-BDNF (1∶500) were diluted in PBS (0.01 M; pH 7.4) with 0.1% Triton-X, 2% NGS and 2% normal donkey serum (Equitech-Bio). After incubation, the sections were rinsed with PBS and incubated with a mixture of FITC-conjugated anti-rabbit IgG (1∶500) and Cy5-conjugated anti-rat IgG (1∶500; Molecular Probes) for nestin/GFAP double labeling, a mixture of FITC-conjugated anti-rabbit IgG (1∶500) and Rhodamine- conjugated anti-chicken IgG (1∶500; Molecular Probes) for nestin/GDNF double labeling, and a mixture of FITC-conjugated anti-rabbit IgG (1∶500) and Rhodamine-conjugated anti-sheep (1∶500; Molecular Probes) for nestin/BDNF double labeling for 2 hours in dark at room temperature. After being rinsed with PBS, the sections were mounted on clean slides with fluorescent mounting medium and covered with coverslips for the examination under the same parameters with laser scan confocal microscope.

### Semi-quantitative analysis

For all the immunofluorescence, the immunoreactivities for nestin, GDNF, BDNF and TH in terms of average grey value by the immunofluorescence staining method were analyzed by software (Metamorph; Universal Imaging). The values were analyzed by statistic program, namely SPSS. And one-way ANOVA (SPSS) was employed to calculate the significant difference between different groups. At least fifteen views from five rats for each group (both sides of striatum and substantia nigra groups) were used for the analysis. For the double labeling, the images of nestin/GFAP, nestin/GDNF and nestin/BDNF double-labeled cells were captured per animal with 3 represented sections [n = 5]. The total cell numbers were counted and presented in mean ± S.E.M.. The nomenclature and description of brain structure were utilized in reference of the atlas of Paxinos and Watson (1986). Controls for the single and double immunofluorescence trials were performed by omission of either the primary or secondary antibodies in the original reaction sequences.

In the Western blotting, optical density of each band was then semi-quantified by Metamorph as well. Control for loading was done in revealing beta-actin proteins using either striatal or nigral tissues of the same animals. Specific bands of 45 kDa were revealed in each of the trial. The optical densities of the bands of BDNF and GDNF were normalized using the optical densities of the bands of beta-actin. The ratios were obtained and statistically analyzed by one-way ANOVA. For ELISA assays, BDNF and GDNF levels were calculated from the standard curve prepared for each plate with SPSS. Data are presented as mean±S.E.M. and compared using one-way ANOVA.

## Results

### The activation of the cell survival mechanisms induced after the administration of 6-hydroxydopamine

**Figure 1:**
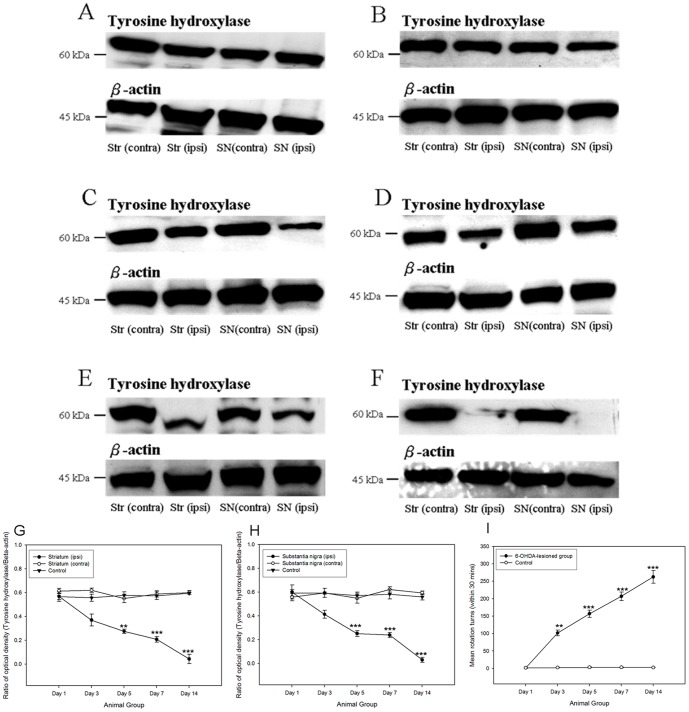
Images showing the TH immunoreactivity of striatum and substantia nigra in 6-hydroxydopamine-lesioned animals amongst different time points under Western blotting. (A) Control; (B) at post-lesion day 1; (C) at post-lesion day 3; (D) at post-lesion day 5; (E) at post-lesion day 7; (F) at post-lesion day 14. The graphs illustrating the time-dependant patterns of tyrosine hydroxylase reactivity in striatum (G) and substantia nigra (H) during post-lesion period. The change in the apomorphine- induced rotation numbers during post-lesion period (I). **p<0.01; ***p<0.001, compared to control group and 6- OHDA- lesioned group (post- lesion day 1).

TH is the key enzyme in the conversion of the amino acid L-tyrosine to dihydroxyphenylalanine (DOPA), which is a precursor of dopamine. It localized with the dopamine cells and acted as an indicator for the degeneration of dopamine cells. In the lesioned animals, immunoreactivity for TH proteins was highly reduced after the 6-OHDA lesion (Figure 1). A significant decrease in the TH expression could be noticed after the post-lesion day 3. Moreover, in the apomorphine- induced rotation test, a graduate increase in the rotation counts (360° turn) was noted.

**Figure 2:**
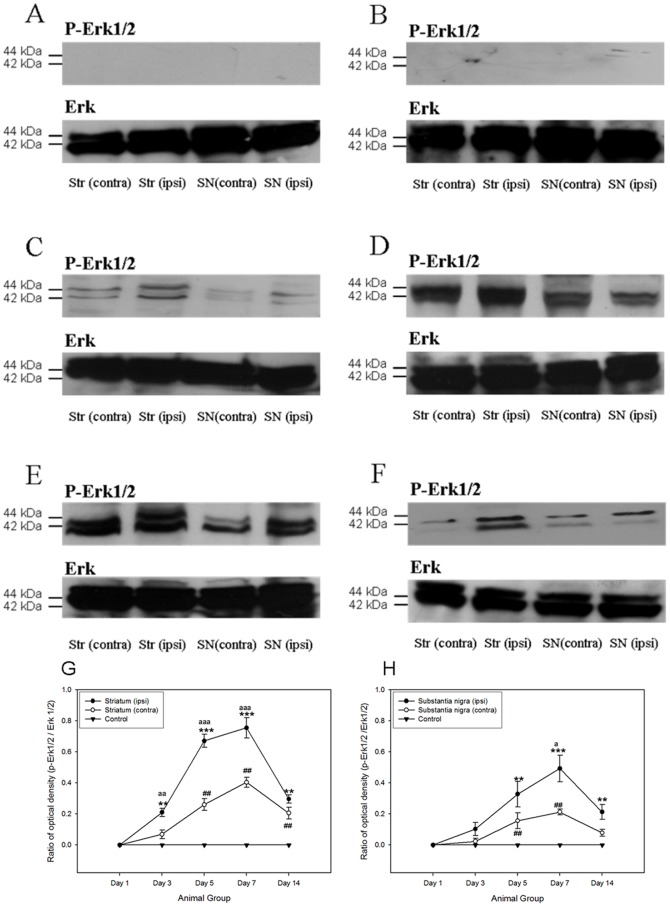
Western Blotting analysis showing that the expression of phosphorylated p44/42 MAPK in both striatum and substantia nigra of the 6- hydroxydopamine-lesioned rats as well as control group amongst different time points. (A) Control; (B) at post-lesion day 1; (C) at post-lesion day 3; (D) at post-lesion day 5; (E) at post-lesion day 7; (F) at post-lesion day 14. The expression patterns of phosphorylated p44/42 MAPK which normalized with p44/42 MAPK in striatum and substantia nigra amongst different post-lesion time points. (G) Striatum; (H) substantia nigra. **p<0.01; ***p<0.001, compared to control group and ipsilateral side of treated group (post- lesion day 1). ^##^ *p*<0.01, compared to control group and contralateral side of treated group (post- lesion day 1). ^a^ *p*<0.05; ^aa^ *p*<0.01; ^aaa^ *p*<0.001, compared to contralateral side of treated group with the respective time point.

**Figure 3:**
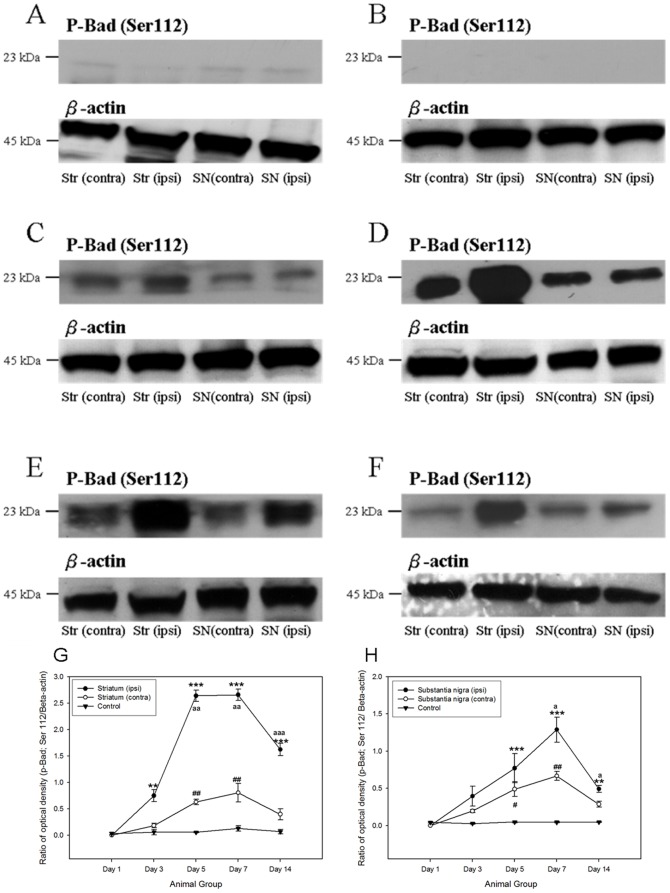
Western Blotting analysis showing that the expression of phosphorylated Bad at Ser 112 in both striatum and substantia nigra of the 6- hydroxydopamine-lesioned rats as well as control group amongst different time points. (A) Control; (B) at post-lesion day 1; (C) at post-lesion day 3; (D) at post-lesion day 5; (E) at post-lesion day 7; (F) at post-lesion day 14. The expression patterns of phosphorylated Bad at Ser 112 which normalized with β-actin in striatum and substantia nigra amongst different post-lesion time points. (G) Striatum; (H) substantia nigra. **p<0.01; ***p<0.001, compared to control group and ipsilateral side of treated group (post- lesion day 1). ^#^ *p*<0.05; ^##^ *p*<0.01, compared to control group and contralateral side of treated group (post- lesion day 1). ^a^ *p*<0.05; ^aa^ *p*<0.01; ^aaa^ *p*<0.001, compared to contralateral side of treated group with the respective time point.

**Figure 4:**
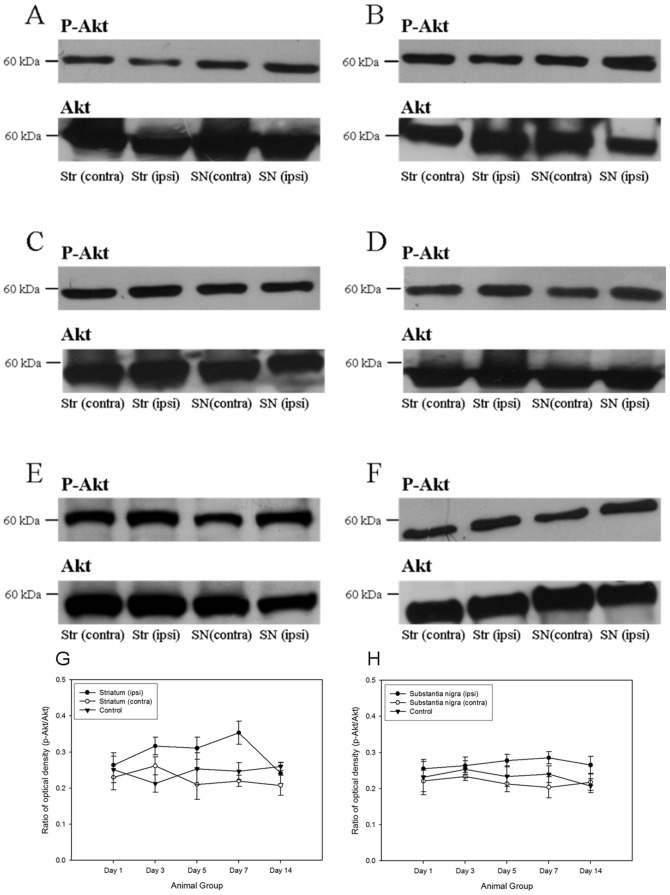
Western Blotting analysi showing the expression of phosphorylated Akt in both striatum and substantia nigra of the 6- hydroxydopamine lesioned rats as well as control group amongst different time points. (A) Control; (B) a post-lesion day 1; (C) a post-lesion day 3; (D) at post-lesion day 5; (E) at post-lesion day 7; (F) at post-lesion day 14. The expression patterns of phosphorylated Akt which normalized with Akt in striatum and substantia nigra amongst different post-lesion time points. (G) Striatum; (H) substantia nigra.

**Figure 5:**
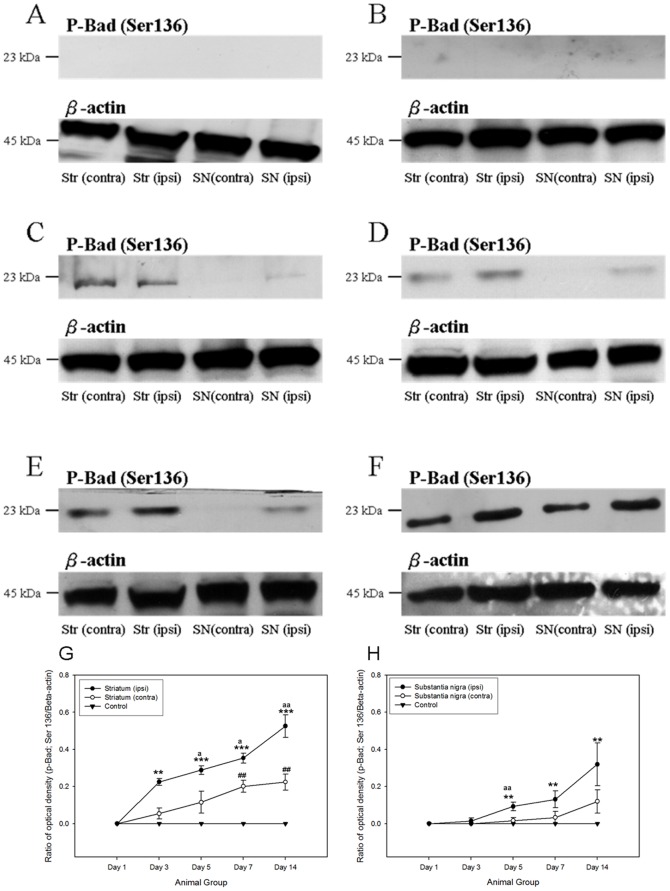
Western Blotting analysis showing that the expression of phosphorylated Bad at Ser 136 in both striatum and substantia nigra of the 6- hydroxydopamine-lesioned rats as well as control group amongst different time points. (A) Control; (B) at post-lesion day 1; (C) at post-lesion day 3; (D) at post-lesion day 5; (E) at post-lesion day 7; (F) at post-lesion day 14. The expression patterns of phosphorylated Bad at Ser 136 which normalized with β-actin in striatum and substantia nigra amongst different post-lesion time points. (G) Striatum; (H) substantia nigra. **p<0.01; ***p<0.001, compared to control group and ipsilateral side of treated group (post- lesion day 1). ^##^ *p*<0.01, compared to control group and contralateral side of treated group (post- lesion day 1). ^a^ *p*<0.05; ^aa^ *p*<0.01; ^aaa^ *p*<0.001, compared to contralateral side of treated group with the respective time point.

A number of anti-apoptotic signaling was initiated after the 6-OHDA-induced degeneration of dopaminergic neurons. The major signaling pathways, including PI3K/Akt cascade [27] and ERK/MAPK cascade, were pivotal for the regulation of cell survival [5], [27], [28], [29], [30], [31], [32], [33]. In both the ipsilateral striatum and substantia nigra of the 6-OHDA-lesioned rats, the phosphorylations of p44/42 MAPK kinase and Akt kinase was increased. Robust expressions of phosphorylated p44/42 MAPK and its downstream target, phosphorylated Bad at Ser 112, were noticed in both ipsilateral striatum and substantial nigra from post-lesion day 3 to day 14, where their immunoreactivities peaked at day 7 (Figure 2 and Figure 3). Phosphorylation of Akt kinase could be observed throughout the period in all groups (Figure 4). No significant changes were found amongst different groups. Interestingly, its downstream target, phosphorylated Bad at Ser 136, illustrated a gradual and significant increase only in the ipsilateral hemisphere of the 6-OHDA-lesioned animals (Figure 5).

### Timely expressions of total brain-derived neurotrophic factor and total glial cell line-derived neurotrophic factor in striatum and substantia nigra of 6-hydroxydopamine lesioned rats

**Figure 6:**
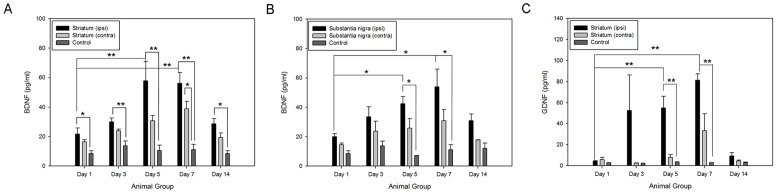
Total endogenous brain derived neurotrophic factor and glial cell-line derived neurotrophic factor were detected in the striatum and substantia nigra of the 6-hydroxydopamine lesioned rats as well as the control group by standard ELISA assays amongst different time points. (A) brain derived neurotrophic factor in striatum; (B) brain derived neurotrophic factor in substantia nigra; (C) glial cell-line derived neurotrophic factor in striatum. *p<0.05; **p<0.01.

The levels of total BDNF and total GDNF protein contents were demonstrated in the ELISA assays (Figure 6). Significant changes in the BDNF and GDNF levels were detected in the ipsilateral hemisphere of the lesioned rats ([n] = 30). Moreover, we observed that the up-regulations of the neurotrophic factors and the cell survival mediators had similar expression patterns. It was well-noted that BDNF and GDNF mediated their neuroprotective effects through the activation of two main cell survival pathways, i.e. PI3K/Akt pathway and ERK/MAPK pathway [28], [29], [30], [31], [32], [33]. In both ipsilateral striatum and substantia nigra, the level of BDNF significantly increased at post-lesioned day 5 (striatum: 55.88±12.87 pg/ml; substantia nigra: 56.25±7.32 pg/ml) and day 7 (striatum: 42.40±6.87 pg/ml; substantia nigra: 53.97±11.95 pg/ml). The GDNF expression was also displayed in the ipsilateral striatum but not in the substantia nigra at post-lesioned day 5 (54.95±10.95 pg/ml) and day 7 (81.18±7.17 pg/ml). The expression patterns of their different molecular forms would be further explored.

### Differential expressions of different molecular forms of neurotrophic factors in nigrostriatal regions after 6-hydroxydopamine lesion

**Figure 7:**
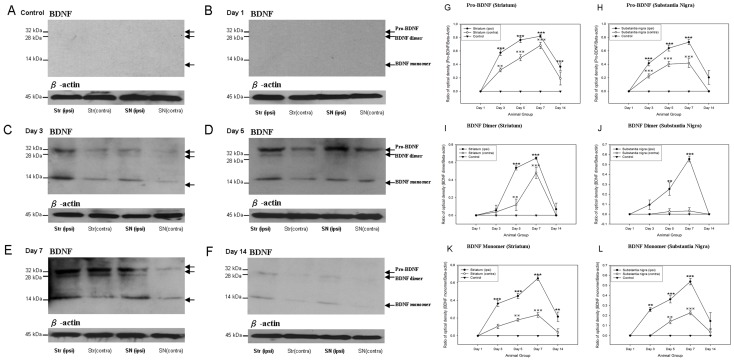
Western Blotting showing that there is an up-regulation of brain-derived neurotrophic factor in both of the striatum and substantia nigra of the 6-hydroxydopamine lesioned rats as well as the control group in different time points. Three bands are observed. They are pro- brain-derived neurotrophic factor (32 kDa), brain-derived neurotrophic factor dimer (28 kDa) and brain-derived neurotrophic factor monomer (14 kDa) respectively (A) Control; (B) Post-lesion day 1; (C) Post-lesion day 3; (D) Post-lesion day 5; (E) Post-lesion day 7; (F) Post-lesion day 14. The expression trends of pro- brain-derived neurotrophic factor (G–H), brain-derived neurotrophic factor dimer (I–J) and brain-derived neurotrophic factor monomer (K–L) of striatum and substantia nigra of the 6-hydroxydopamine lesioned rats as well as the control group during different time points are illustrated in the graphs. **p<0.01; ***p<0.001; 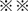 *p*<0.01; 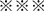 *p*<0.001.

Three bands of BDNF proteins were observed in the Western Blotting analysis (Figure 7). These three bands were Pro-BDNF (32 kDa), BDNF dimer (28 kDa) and BDNF monomer (14 kDa) respectively (40). Pro-BDNF is the precursor for synthesis of mature forms of BDNF (14 kDa). Cleavage of pro-BDNF by the serine protease plasmin system formed BDNF monomer. Monomers of BDNF were unable to bind to the TrkB receptor until the dimerization of two BDNF monomers (28 kDa) [34]. In both the striatum and the substantia nigra, expressions of pro-BDNF and BDNF monomer were observed at post-lesion day 3 while the expression of BDNF dimer was only started at post-lesion day 5 (Figure 7A–F). The levels of pro-BDNF increased significantly from post-lesion day 3 to day 7 and declined at post-lesion day 14 (Figure 7G–H). Then the peak of BDNF dimer and BDNF monomer expressions were found to be at post-lesion day 7 (Figure 7I–L). Interestingly, in the contralateral striatal and nigral tissues of the 6-OHDA-lesioned rats, pro-BDNF and BDNF forms were also up-regulated with an insignificant amount. (Figure 7G–L). The only exception was that no significant increase in BDNF dimer was detected in the contralateral tissues (Figure 7J). Furthermore, the expressions of pro-BDNF and BDNF forms were lesion-specific (Figure 7G–L).

**Figure 8:**
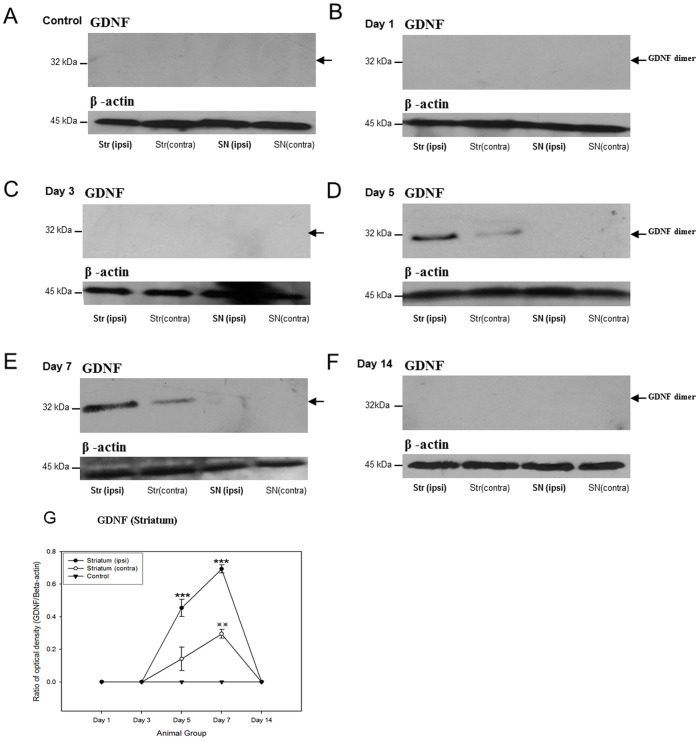
Western Blotting analyses showing that there is an up-regulation of glial cell-line derived neurotrophic factor both the striatum and substantia nigra of the 6-hydroxydopamine lesioned rats as well as the control group in different time points. (A) Control; (B) Post-lesion day 1; (C) Post-lesion day 3; (D) Post-lesion day 5; (E) Post-lesion day 7; (F) Post-lesion day 14. The expression trends of glial cell-line derived neurotrophic factor of striatum and substantia nigra of the 6-hydroxydopamine lesioned rats as well as the control group during different time points are illustrated in G. ***p<0.001; 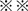 *p*<0.01.

A single 32 kDa band of GDNF homodimer was observed [35] in the blots. GDNF protein was found to express at post-lesion day 3 and peaked at post-lesion day 7 (Figure 8). Lower levels of GDNF proteins were also detected in the contralateral striatum (Figure 8). This observation was consistent with the above analysis that no GDNF protein was observed in the substantia nigra.

### Reactive astrocytes are potential candidates to offer the endogenous repair mechanisms by promoting the expression of the neurotrophic factors

**Figure 9:**
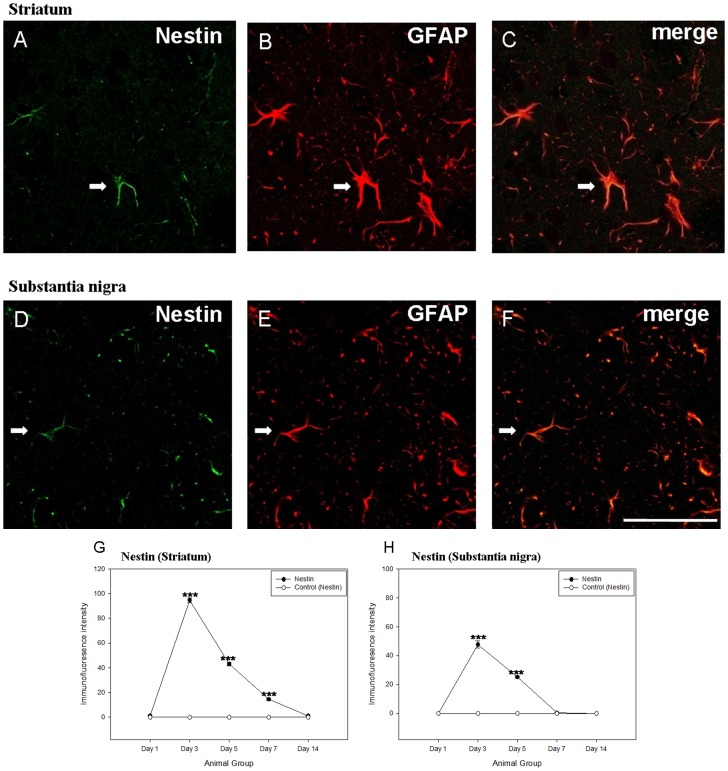
Laser scanning confocal microscopy showing (A–C) nestin, glial fibrillary acid protein, Nestin/glial fibrillary acid protein immunoreactivity in the striatum (as indicated by arrow) and (D–F) substantia nigra (as indicated by arrow) of the 6-hydroxydopamine-lesioned day 3 rat. Co-location of nestin with glial fibrillary acid protein can be observed. Scale bar: 25 µm. Comparisons of the average grey value of various labeling in both of the striatum and substantia nigra. The time-dependant patterns of tyrosine hydroxylase reactivity in striatum (G) and substantia nigra (H).

**Figure 10:**
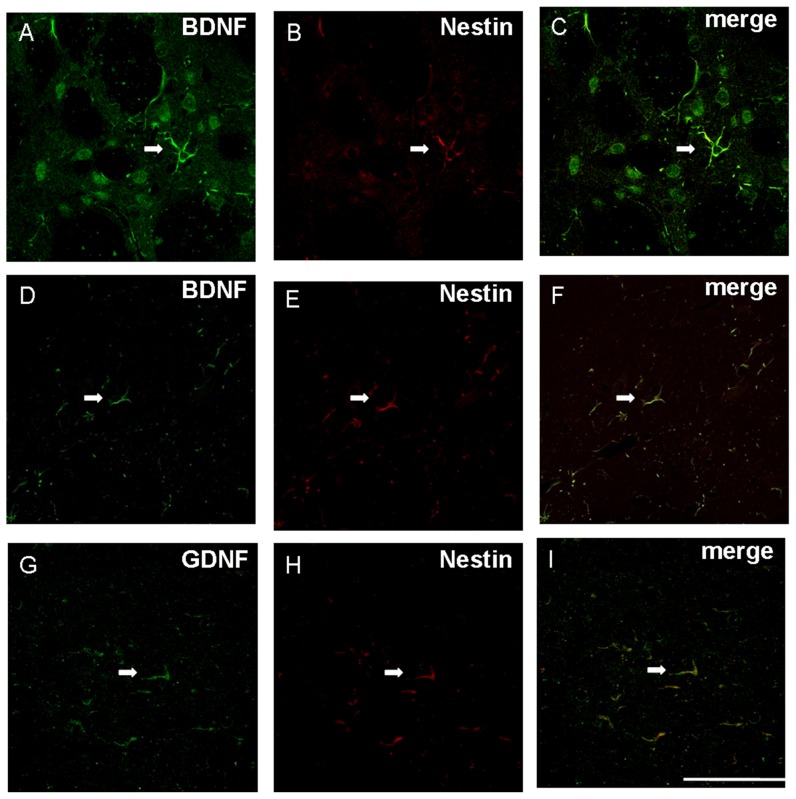
The nestin immunoreactive cells is found to be overlapped with that of brain-derived neurotrophic factor immunoreactive cells in both striatum and substantia nigra of the 6-hydroxydopamine lesioend rats while that overlapped with that of glial cell-line derived neurotrophic factor immunoreactive cells in the striatum. a–c show an astrocyte (arrow) that displays brain-derived neurotrophic factor, Nestin, brain-derived neurotrophic factor/Nestin immunoreactivity in striatum; d–e show an astrocyte (arrow) that displays brain-derived neurotrophic factor, Nestin, brain-derived neurotrophic factor/Nestin immunoreactivity in substantia nigra; g–i show an astrocyte (arrow) that displays glial cell-line derived neurotrophic factor, Nestin, glial cell-line derived neurotrophic factor/Nestin immunoreactivity in striatum. Scale bar: 50 µm.

We previously reported that reactive astrocytes with nestin were re-expressed in the 1-methyl-4-phenyl-1,2,3,6-tetrahydropyridine (MPTP)- lesioned mice [29]. In the present study, nestin immunoreactivity was also re-appeared in the astrocytes of both the ipsilateral striatum and substantia nigra after the 6-OHDA administration (Figure 9). Furthermore, a specific expression pattern could be revealed in the lesioned rats ([n] = 30; Figure 9). A low level of nestin was detected at post-lesion day 1. Its expression then reached the peak at post-lesion day 3 and decreased after post-lesion day 5. The expression was hardly observed in both striatum and substantia nigra at post-lesion day 14. In the meanwhile, BDNF and GDNF were co-localized with these nestin-positive reactive astrocytes. Quantification data showed that 87.7±7.2% and 74.2±4.9% expressed BDNF in the nestin-immunoreactive astrocytes of striatum and substantia nigra respectively at post-lesion day 5, in which nestin immunoreactivity was still strong (Figure 10). Moreover, 83.7±5.8% and 62.1±4.6% exhibited nestin in the BDNF-immunoreactive astrocytes of striatum and substantia nigra at post-lesion day 5 respectively. A low level of BDNF immunoreactivity was expressed by perikarya of neurones near to BDNF immunoreactive reactive astrocytes in the striatum but not in the substantia nigra (Figure 10). On the other hand, quantification data showed that 46.1±7.3% expressed GDNF immunoreactivity in the nestin-immunoreactive astrocytes of striatum whilst 98.2±1.3% of GDNF-immunoreactive cells expressed nestin immunoreactivity (Figure 10). These data suggested that reactive astrocytes might be responsible for the release of the neurotrophic factors and thus provoke the self-neuroprotective mechanisms.

## Discussion

**Figure 11:**
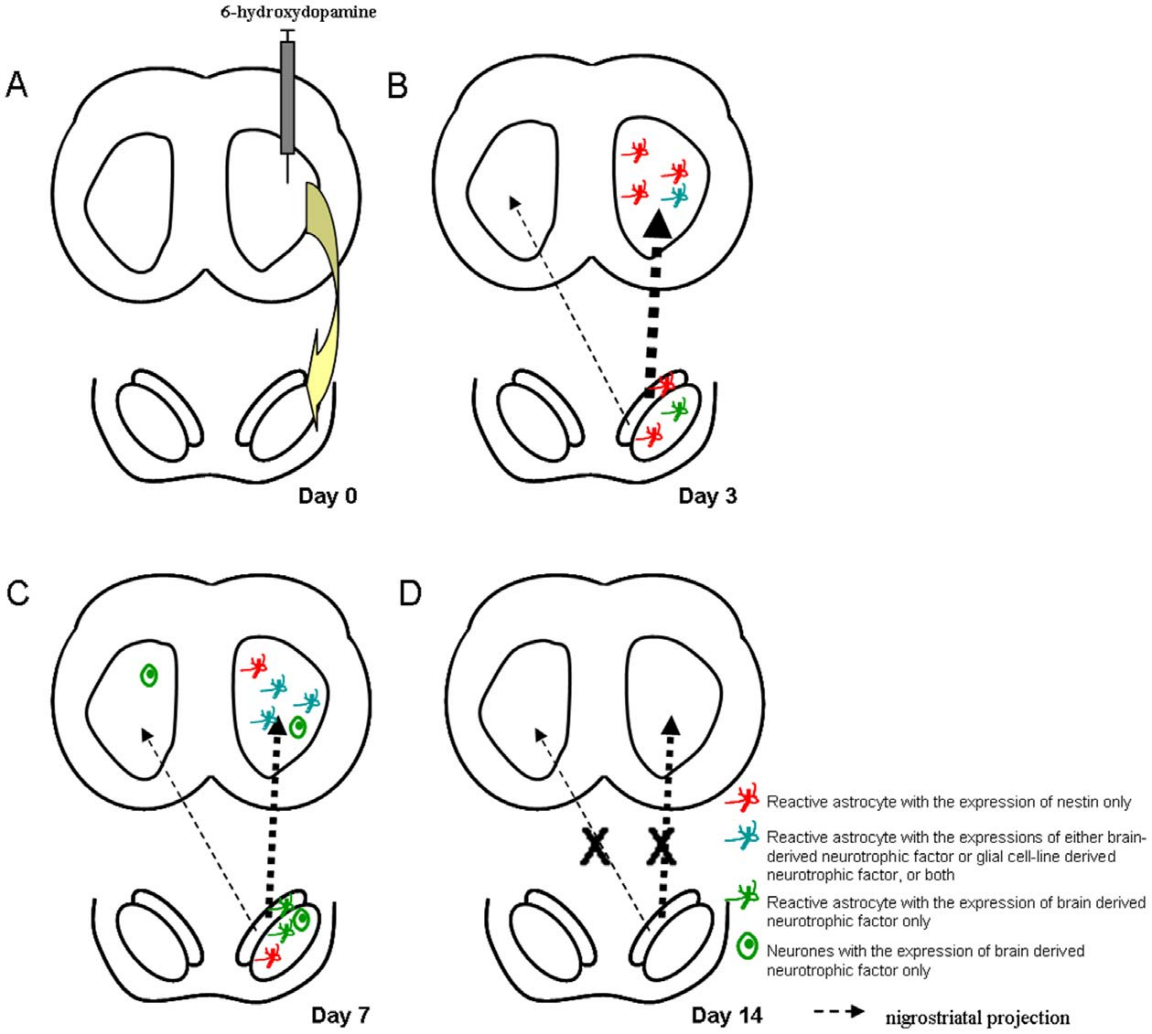
(A) Unilateral administration of 6-hydroxydopamine in striatum induces a significant degeneration of ipsilateral substantia nigra through the nigrostriatal pathway. (B) The expression of nestin-immunoreactive reactive astrocytes is found to peak at post-lesion day 3. Low levels of expression of brain derived neurotrophic factor in reactive astrocytes are found in the ipsilateral striatum as well as the ipsilateral substantia nigra. Expression of glial cell-line derived neurotrophic factor is found in the ipsilateral striatum only. (C) The expression of nestin in reactive astrocytes drops to a low level in both ipsilateral striatum and ipsilateral substantia nigra at post-lesion day 7. However, the expressions of the brain-derived neurotrophic factor and glial cell-line neurotrophic factor in the reactive astrocytes are found to be peaked at the same period. The expression of brain-derived neurotrophic factor can be found in the neurones of the ipislateral striatum and substantia nigra, as well as the contralateral striatum, but in a small amount. (D) No expressions of nestin, brain derived neurotrophic factor and glial cell-line derived neurotrophic factor immunoreactivities are found in the striatum and substantia nigra at post-lesion day 14.

The present results have demonstrated for the first time that astrocytes exhibit an endogenous self-repairing mechanism via release of BDNF and/or GDNF during very early stages of rat Parkinsonism of 6-OHDA-leisoned rats. Importantly, we have found there is a specific sequence of events for these possible self repair mechanisms (Figure 11). These events happen at extremely early stages of degeneration. It is often missed in animal models and is impossible to be discovered in patients with Parkinson's disease. At early post-lesion day 3, levels of TH in the ipsilateral striatum started to lower. Nestin immunopositive reactive astrocytes started to appear and some of them were found to display BNDF and/or GDNF. The number of reactive astrocytes displayed BDNF and GDNF then reached the peak at post-lesion day 7. Neuronal expression of BNDF was also found at this stage. At post-lesion day 14, no more reactive astrocytes were then observed in striatum and the transient self-repair events were curtained. Interestingly, GDNF is not expressed by reactive astrocytes in the substantia nigra. GDNF is strongly presented in the striatal medium spiny neurones that receive the dopaminergic inputs from the substantia nigra [36]. GDNF also supports the embryonic development and postnatal survival of dopaminergic neurones in the substantia nigra and their axonal terminals in the striatum [31], [32], [36]. Reactive astrocytes also appeared in the ipsilateral substantia nigra at post-lesion day 3 and only some of them displayed BDNF. The pattern of appearance of BDNF in reactive astrocytes and neurones in the substantia nigra followed that of striatum and the peak of BDNF expression was found at post-lesion day 7. At post-lesion day 14, the self-repair events were also curtained in the substantia nigra. In addition, based on the above findings, ERK/MAPK pathway was most likely involved in the endogenous neuroprotective mechanisms, particularly in early stages of rat Parkinsonism. Phosphorylation of p44/42 MAPK kinase as well as it downstream target, Bad (Ser 112), could be observed significantly during the studied post-lesion period, i.e. from post-lesion day 1 to post-lesion day 14, in response to 6-OHDA toxin.

Among families of neurotrophic factors, BDNF and GDNF are found to be the major factors that protect dopaminergic neurons [28], [29], [30], [33], [37] and associated with neuronal survival, neurite outgrowth and neuronal functions [35], [38]. Expressions of BDNF and GDNF are found to be abundant in striatum during embryonic and early postnatal development [29]. However, in the normal adult rats, BDNF exists at a low level while GDNF is undetectable [5]. The neuroprotective effect of BDNF and GDNF probably is mediated by inducing phosphorylations of several signaling proteins which inhibits the apoptotic signaling from the brain insults and toxins [28]. BDNF binds to Trk B receptors and initiates a number of anti-apoptotic signaling pathways including phorphtidylinositol 3-kinase (PI3K)/Akt pathway [27], [39]. Activation of these pathways promotes Bcl-xL/Bcl-2-associated death promoter (BAD) at Ser 136 and at Ser 112. These phosphorylations lead to inhibition of the apoptotic pathways and thus enhance cell survival [28], [40], [41], [42], [43], [44], [45], [46], [47], [48]. On the other hand, GDNF binds to RET, a tyrosine kinase receptor and then induces the autophosphorylation of RET [27], [28], [39], [40], [41], [42], [43], [44], [45], [46], [47], [49]. This phosphorylation also activates the PI3K/Akt pathway [28], [49]. Release of BDNF and GDNF from reactive astrocytes are therefore able to promote survival of the degenerating dopaminergic neurones after exposure to 6-OHDA.

Many intensive researches were done both preclinically and clinically on the efficacy of the neurotrophic factors as the treatment of Parkinson's disease. Previous studies in animals and in clinical trials have indicated that delivery of GDNF and BDNF have provided certain degrees of efficacy in treatments of Parkinsonian patients [31], [32], [36]. However, clinical trials of GDNF infusions on 34 patients failed recently due to unexpected immune responses in approximately half of the patients and some resulted in mortality. It is still not clear whether these unfortunate events are deal to the infusion of GDNF or some other causes including heart attack [50], [51]. Despite the fact that current clinical trials are underway employing infusion of BDNF or gene transfection of neurturin, there is often a risk in administration of exogenous growth factors into the nervous systems [31], [32], [36]. Our findings are important because the results indicate that there is an endogenous process that can release endogenous BNDF and GDNF from reactive astrocytes upon onset of Parkinson's disease. These demonstrate directly that the nervous system has the ability to exercise self repair at least at the very early beginning of the degenerative process. If the repair mechanisms can be maintained for longer time period, it can have significant clinical implications. The rationale for putting exogenous BDNF and GDNF may need to be re-visited.

In addition to release of BDNF and GDNF, previous studies have shown reactive astrocytes facilitated neuroprotective and neurotrophic effect on dopaminergic neurons [52], [53], [54], [55]. However, a key issue here is that it is unknown how an astrocyte turns reactive and the triggering mechanism of the nestin re-expression. It is suggested to trigger by oxidative stress mechanisms [56], [57], [58] and the release of triggering factors such as the excitatory amino acids derived from the damaged dopaminergic neurons [29]. Others have hypothesized that astrocytic network is linked by gap junctions and direct information about neuronal damage or reduced neuronal activity from the site of damage can be passed from one area to the others [59], [60]. Degeneration of neurones will also interrupt the glial communication and lead to a decrease the gap junctions amongst the astrocytes and causes the astrocytes turn reactive [58], [60].

In reactive astrocytes, astrocytic Ca^2+^ oscillations have been well documented [61], [62], [63]. When astrocytes turn reactive, Ca^2+^ are released from internal stores of astrocytes with additional influx of extracellular Ca^2+^ through voltage-gated channel, and thus result in a transient Ca^2+^ oscillations [61], [64]. Ca^2+^ regulated the transcriptions of BDNF and GDNF through the cAMP response element binding (CREB)-dependent mechanism [64], [65] and mitogen-activated protein (MAP) kinase-dependent and -independent pathways [66]. Increases in intracellular Ca^2+^ is known to enhance the synthesis of pro-BNDF and pro-GDNF, and therefore the increased Ca^2+^ levels in reactive astrocytes then trigger the release of BDNF as well as GDNF. In addition, Ca^2+^ is required for complex formation between RET and GDNF to form signal transducing complex [67] so as to activate the anti-apoptotic pathways.
